# “I couldn’t buy the items so I didn’t go to deliver at the health facility” Home delivery among rural women in northern Ghana: A mixed-method analysis

**DOI:** 10.1371/journal.pone.0230341

**Published:** 2020-03-12

**Authors:** Michael Boah, Timothy Adampah, Baiming Jin, Siyuan Wan, Abraham Bangamsi Mahama, Dalia Hyzam, Caselia Akiti

**Affiliations:** 1 Center for Endemic Disease Control, Chinese Center for Disease Control and Prevention, Harbin Medical University, Harbin, Heilongjiang, China; 2 Ghana Health Service, Private Mail Bag Bolgatanga, Upper East Region, Ghana; 3 Education, Culture and Health Opportunities (ECHO) Research Group International, Aflao, Ghana; 4 Department of Preventive Medicine, Qiqihar Medical University, Qiqihar, Heilongjiang, China; 5 United Nations Children’s Fund, United Nations House, Abuja, Nigeria; 6 Department of Children’s and Adolescent Health, School of Public Health, Harbin Medical University, Harbin, China; University of Oxford, UNITED KINGDOM

## Abstract

**Background:**

Maternal mortality remains a major challenge to health systems in low and middle-incoming countries. Some pregnant women develop potentially life-threatening complications during childbirth. Therefore, home delivery is a precursor for maternal mortality. In this study, we aimed at not only estimating the percentage of deliveries occurring at home and examining the factors associated with home delivery, but we also explored the reasons for home delivery among women in rural Ghana.

**Methods:**

The study was conducted among mothers with delivery experience in selected communities in the Builsa South district located in the Upper East Region of Ghana. Both quantitative and qualitative data were collected using semi-structured questionnaires and Focus Group Discussion (FGD) guide respectively. A total of 456 mothers participated in this study. Regression models were used in the quantitative analysis whereas a thematic analysis approach was used to analyze the qualitative data.

**Results:**

Of the 423 mothers in the quantitative research, 38.1% (95% CI: 33.5–42.8) delivered their index child at home. In adjusted analysis, women who were not exposed to information (AOR = 13.64, p<0.001) and women with 2 (AOR = 4.64, p = 0.014), 3 (AOR = 4.96, p = 0.025) or at least 4 living children (AOR = 9.59, p = 0.001) had higher odds of delivering at home. From the qualitative analysis, the poor attitude of nurses (midwives), lack of, and cost of transportation, cost of delivery kits, and traditional beliefs and practices were cited as reasons for home delivery.

**Conclusion:**

Despite the government’s efforts to provide free maternal care services to women in Ghana, a significant proportion of rural women still deliver at home due to other ‘hidden costs’. Addressing poor staff attitude, transportation challenges, and negative traditional beliefs and practices through awareness creation may contribute to improving health facility delivery by rural pregnant women in Ghana.

## Introduction

The death of a woman from a pregnancy-related cause is a very sad event because the necessary interventions needed to prevent maternal deaths are well known [1]. Globally, more than 95% of the maternal deaths that occurred in 2017 were from low and middle-income countries (LMICs), the highest proportion occurred in sub-Saharan Africa [2]. The maternal mortality ratio in Ghana currently stands at 308 deaths per 100,000 live births. Though this figure is relatively lower compared to the number of maternal deaths that occurred over a decade ago, it still remains an issue of concern if the Target 3.1 of the Sustainable Developments Goals (SDGs) has to be met by 2030 [1].

Access to maternal health services, including skilled birth attendant (SBA), has been proven to be important in reducing maternal deaths. The WHO recommends the presence of an SBA during delivery because complications can arise anytime during labor and delivery [3]. However, physical access and finance have long been identified as major barriers to the use of health services by women in LMICs [4].

To address these barriers in Ghana, the government implemented and scaled-up the Community-based Health Planning and Services (CHPS) initiative nation-wide in 2002, and followed up with the free maternal health care policy and the national health insurance scheme (NHIS). The CHPS initiative was established from the “Navrongo experiment”, known as the Community Health and Family Planning (CHFP) project, to improve geographic access to quality health care and family planning services while empowering local communities to take greater control over their health [5,6]. On the other hand, the free maternal health care policy provided a free ‘maternal health package’ which covered maternal health services women receive from the time of pregnancy to three months after delivery [7]. The aim of this initiative was to reduce financial barriers to accessing maternity services.

The user-fees exemption policy (or NHIS) that has been in place for the past decade or so has contributed significantly to reducing out-of-pocket expenditure for maternal health services received from the public health facilities in Ghana [8,9]. An evaluation of the policy in the Central and Volta regions in Ghana revealed an increase in the percentage of health-facility deliveries post-implementation [10]. The findings further demonstrated that the greatest increases in skilled deliveries occurred among the impoverished. At the national level, antenatal care (ANC) utilization and skilled delivery have improved significantly since 2003. For instance, the percentage of deliveries occurring in a health facility has increased from 46% in 2003 to 73% in 2014; the percentage of births attended by an SBA has increased from 47% to 74% during the same period [11]. These observations support the argument that the implementation of the user-fees exemption policy has greatly eliminated financial barriers to access. However, reports have emerged more recently that some beneficiary households of the user-fees exemption policy in Ghana still suffer some level of catastrophic costs in receiving primary health care in public health institutions [9].

Consistent with this development, despite the nearly universal coverage in prenatal care utilization, a quarter of pregnant Ghanaian women shun the health facility in favor of the home for a delivery, the situation being worse in the rural areas (59% versus 90% in urban areas) [11]. Home deliveries with traditional birth attendants (TBAs) are associated with increased risk of infection, and increased risk of mortality [12,13]. In fact, about half of the neonatal deaths recorded in LMICs occur at home without the presence of an SBA [14]. Elsewhere, home delivery has been associated with lower cognitive function among individuals later in adulthood [15].

The available literature in Ghana has identified that women’s choice of place for delivery is shaped by individual-level factors such as maternal age, education, religion, ANC utilization, and maternal knowledge [16–18]. However, the factors which influence women’s choice of delivery place operate at various levels including household and community [19–21]. Identifying only factors at the individual level dwarfs other factors that are directly or indirectly influencing women’s decisions on the choice of delivery place at the individual level. Therefore, using both quantitative and qualitative methods, this study sought to determine not only the prevalence of home delivery and the factors associated with delivery at home but also to explore the reasons behind women’s use of the home over the health facility for delivery under the free maternal health policy in a predominantly rural district in northern Ghana.

## Material and methods

### Study design and setting

This study was cross-sectional in design. Both quantitative and qualitative methodologies to research were employed. The survey was carried out in the Builsa South district located in the Upper East Region of northern Ghana. The Builsa South district was selected because the available data from the district’s 2014 annual report showed that health facility delivery had declined by 21.2% from 67.4% in 2012 to 46.2% in 2014 (unpublished report). The district is predominantly rural with a dispersed settlement system. At the time of the survey, the district had an estimated population of 38, 298 people being served by 17 public health facilities including 3 health centres and 14 CHPS zones. These health facilities are unevenly distributed among 6 sub-districts. There were no private maternity homes in the district. Women in the reproductive age (15–49 years) form 24% (9,192) of the total population of the district [22].

### Sampling and selection of study participants

#### Quantitative study

All women aged 15–49 years in the selected communities were eligible to participate in this study. However, a total sample of 423 postpartum women aged 15–49 years who delivered in the past six months preceding the survey was included in the quantitative study. The sample size was estimated using the single population proportion formula [23].

Multi-stage sampling procedures were followed to select study communities and participants. In the first stage, one community (Bachongsa, Samsa, Zamsa, Wiesi central, Gbenaasa and Fumbisi central) in each of the six sub-districts was randomly selected. The total sample of 423 women was proportionally allocated to the selected communities. The second stage involved the selection of households and the women for the interviews. A sampling frame of households with eligible women was developed using community filariasis registers alongside child welfare clinic registers for complete and up-to-date information on eligible households. Using systematic random sampling, the number of households required in each community was selected. Only one woman in a selected household was interviewed. If a household had more than one eligible woman, random sampling (lottery method) was used to select one for the interview.

#### Qualitative study

Four of the six selected communities were randomly identified for the qualitative study. Focus Group Discussions (FGDs) were conducted with reproductive women who did not participate in the quantitative study. The FGDs were preferred over in-depth interviews because of the comparative advantages [24–26]. Participants for the FGDs were purposively selected based on their age and experiences with delivery care [27]. A gatekeeper was informed by the research team about the characteristics of the women required for the FGDs. A total of 33 women participated in the FGDs. Each FGD had an average of 8 participants.

### Data collection and analysis

Quantitative data were collected using structured questionnaires (see S1 File). The information gathered covered women’s demographic and obstetric characteristics, ANC use, and place of delivery for her most recent pregnancy. Data collectors with at least secondary education and experience with data collection were recruited to assist in the data collection process. Information in the qualitative research was collected using an FGD guide (see S2 File). A two-day training session was organized for the data collectors by members of the research team to equip them with interviewing skills. The data collection tools were translated into the local language (Buli) during the training period and pretested in communities in a neighboring district (Builsa North district) with similar characteristics to the study district before the actual data collection. Ambiguous questions were revised. The data collectors were supervised throughout the data collection period. The FGDs were facilitated by a moderator and a note-taker.

Completed questionnaires were checked daily for completeness and consistency. Data were entered in EpiData Entry Client version 2.06.20 (EpiData Association, Denmark), and the dataset exported to SPSS version 20 for data cleaning and analysis. The dependent variable was the place of delivery. It is a binary variable with “No” indicating deliveries which took place in a health facility and “Yes” for deliveries which took place at home or outside the health facility. The outcome of interest was home delivery. Maternal age, education, religious affiliation, employment status, exposure to information, health insurance enrollment, gestational age of pregnancy at ANC registration, and the number of ANC follow-up visits made before delivery were included in the analysis as independent variables. Both univariable and multiple logistic regression models were used to examine the association between the independent variables and the dependent variable. Only independent variables returning p-values less than or equal to 0.05 in the univariable logistic regression analysis were fitted into the multiple logistic regression to examine their independent associations. A p-value of less than 0.05 was considered statistically significant in the final model.

The FGDs were tape-recorded and detailed notes were taken during the discussions. The recordings were transcribed verbatim by two research assistants. The data were analyzed using the thematic analysis approach (see S3 File) proposed by Braun & Clark [28]. This technique was considered appropriate for this study because it is flexible to use, and provides a rich analysis of the data generated. We ensured that there was enough data to support each potential theme.

### Data quality and control

Threats to data quality and validity were addressed by collecting rich data in the research process itself. The data collectors and field supervisors received training on the data collection process during which simulation interviews were conducted. The structured questionnaire and discussion guide were assessed and modified by experts in reproductive health research from the School of Public Health, University of Ghana. The data collection tools were pre-tested and modified before actual data collection. All the FGD sessions were tape-recorded, transcribed verbatim by two independent research assistants and checked for consistency. No significant differences were observed. Detailed field notes were taken during the discussions and feedback was solicited from participants by sharing the detailed notes at the end of each discussion for a consensus between the moderator and the participants on the interpretation of participants’ opinions.

### Ethical considerations

The study received ethical approval from the ethics review committee (ERC) of the Ghana Health Service. Written informed consent was obtained from all participants after information about the study, including, the potential risks and benefits, was communicated. In the FGDs, permission was sought from participants before the discussions were audio-recorded. The anonymity of all study participants was ensured by removing all personal identifiers before data analysis.

## Results

### Quantitative findings

#### Socio-demographic and obstetric characteristics of the study participants

**Fig 1:**
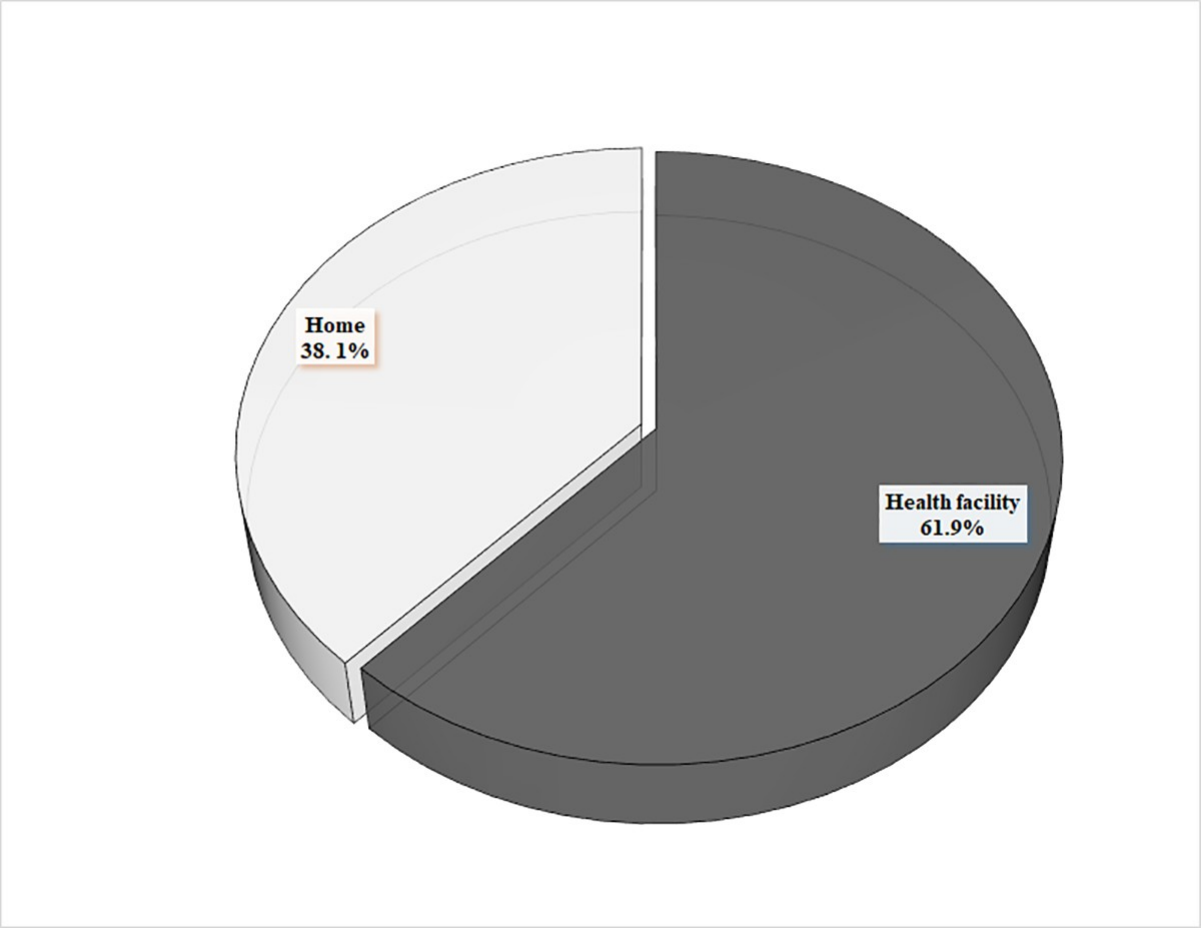
The distribution of women aged 15–49 years by place of recent delivery (N = 423 women).

The socio-demographic and obstetric characteristics of the study participants are presented in Table 1. Briefly, the mean age of the participants was 29.2±7.4 years (Range: 15–49 years). A greater proportion of the participants were Christians (62.2%), employed (81.3%), in a union (98.1%) and registered with the NHIS (83.0%). Regarding their obstetric characteristics, 418 (98.8%) of the 423 women used prenatal services at least once during the recent pregnancy, of which 56.9% initiated their first ANC visit after 3 months of pregnancy, and 67.9% made at least 4 ANC follow up visits before delivery. Of the total sample, 91.5% were exposed to delivery care information during their recent pregnancy, of which the most frequently mentioned source was the health worker (73.4%), followed by the radio (32.6%), community health volunteers (27.6%), and television (6.5%) (Table 1). Of the 423 women in this study, 161 reported delivering the index child at home giving a prevalence of 38.1% (95% CI: 33.5–42.8) for home delivery in the study district (Fig 1).

**Table 1:** Socio-demographic and obstetric characteristics of the study participants (N = 423 unless indicated).

Characteristic	Number	Per cent
**Age (Years)**		
Mean age (Mean ± Standard deviation)	29.2±7.4	
15–24	131	31.0
25–34	179	42.3
35–49	113	26.7
**Maternal education**		
No formal education	172	40.7
Basic education	204	48.2
Secondary or higher	47	11.1
**Employment status**		
Unemployed	79	18.7
Employed	344	81.3
**Marital status**		
Not in a union	8	1.9
In a union	415	98.1
**Ethnicity**		
Builsa	413	97.6
Others^a^	10	2.4
**Religious affiliation**		
African traditional	115	27.2
Islam	45	10.6
Christian	263	62.2
**Health insurance enrollment**		
Registered	351	83.0
Not registered	72	17.0
**ANC use during recent pregnancy**		
Yes	418	98.8
No	5	1.2
**Gestational age of pregnancy at first ANC booking (N = 418)**		
First trimester	180	43.1
Second trimester or over	238	56.9
**Number of ANC visits before delivery (N = 418)**		
Less than 4	134	32.1
4 or more visits	284	67.9
**Number of living children**		
1	87	20.6
2	101	23.8
3	73	17.3
4 or more	162	38.3
**Exposure to information**		
Exposed	387	91.5
Not exposed	36	8.5
**Source of information on delivery care**^b^ **(N = 387)**		
Radio	126	32.6
Television	25	6.5
Health worker	284	73.4
Community health volunteers	107	27.6

^a^Others comprised of Mamprusi (4), Kassena (4) and Sissala (2)

^b^Multiple responses were allowed

### Factors associated with home delivery among women aged 15–49 in rural northern Ghana

The results of the univariable logistic regression are presented in Table 2. The findings showed that odds of home delivery was higher among women in the age group of 25–34 years (OR = 2.72, 95% CI: 1.60–4.62, p<0.001) and 35–49 years (OR = 5.95, 95% CI: 3.45–10.57, p<0.001) compared with women in the age group of 15–24 years; higher among women with no formal education (OR = 17.70, 95% CI: 6.07–51.59, p<0.001) and women with basic level education (OR = 3.49, 95% 1.19–10.21, p = 0.022) compared with women with secondary or higher level of education; higher among women not exposed to information on delivery care (OR = 3.19, 95% CI: 1.57–6.50, p = 0.001) compared with women exposed to information on delivery care; and, higher among women not registered with the NHIS (OR = 3.38, 95% CI: 1.99–5.72, p<0.001) compared with women who were registered. Also, we noted that the odds of delivering at home was higher for women with two or more children compared with women with one living child.

**Table 2:** Characteristics associated with home delivery among women aged 15–49 in rural northern Ghana (N = 423 unless indicated).

Characteristic	Number (n)	Home delivery		Crude Odds ratio	P-value
		No (%)	Yes (%)	OR(95% CI)	
**Age (Years)**					
15–24	131	80.9	19.1	Reference	
25–34	179	60.6	39.4	2.72(1.60–4.62)	<0.001
35–49	113	41.6	58.4	5.95(3.35–10.57)	<0.001
**Maternal education**					
No formal education	172	37.8	62.2	17.70(6.07–51.59)	<0.001
Basic education	204	75.5	24.5	3.49(1.19–10.21)	0.022
Secondary or higher	47	91.5	8.5	Reference	
**Employment status**					
Unemployed	79	38.0	62.0	Reference	
Employed	344	67.4	32.6	0.30(0.18–0.49)	<0.001
**Marital status**					
Not in a union	8	75.0	25.0	Reference	
In a union	415	61.7	38.3	1.86(0.37–9.34)	0.449
**Ethnicity**					
Builsa	413	61.7	38.3	1.45(0.37–5.67)	0.597
Others	10	70.0	30.0	Reference	
**Religious affiliation**					
African traditional	115	39.1	60.9	Reference	
Islam	45	66.7	33.3	0.32(0.16–0.66)	0.002
Christian	263	71.1	28.9	0.26(0.16–0.41)	<0.001
**Exposure to information**					
Exposed	387	64.3	35.7	Reference	
Not exposed	36	36.1	63.9	3.19(1.57–6.50)	0.001
**Health insurance enrollment**					
Registered	351	67.0	33.0	Reference	
Not registered	72	37.5	62.5	3.38(1.99–5.72)	<0.001
**Gestational age of pregnancy at first ANC booking (N = 418)**					
First trimester	180	96.1	3.9	0.02(0.01–0.05)	<0.001
Second trimester or over	238	37.4	62.6	Reference	
**Number of ANC visits before delivery (N = 418)**					
Less than 4	134	14.2	85.8	Reference	
4 or more visits	284	85.6	14.4	0.03(0.02–0.05)	<0.001
**Number of living children**					
1	87	86.2	13.8	Reference	
2	101	68.3	31.7	2.90(1.38–6.07)	0.005
3	73	68.5	31.5	2.88(1.31–6.30)	0.008
4 or more	162	42.0	58.0	8.64(4.36–17.13)	<0.001

However, we noted lower odds of home delivery among women who were employed (OR = 0.30, 95% CI: 0.18–0.49, p<0.001) compared with those who were unemployed; women who identified with Islam (OR = 0.32, 95% CI: 0.16–0.66, p = 0.002) or Christianity (OR = 0.26, 95% CI: 0.16–0.41, p<0.001) compared with women who identified with African traditional religion; women who booked their first ANC visit within 3 months of pregnancy (OR = 0.02, 95% CI: 0.01–0.05, p<0.001) compared with women who booked after 3 months gestational period; and, women who made at least 4 ANC follow-up visits before delivery (OR = 0.03, 95% CI: 0.02–0.05, p<0.001) compared with women who made less than 4 ANC follow-up visits before delivery (Table 2).

### Factors independently associated with home delivery among women aged 15–49 in rural northern Ghana

In the adjusted analysis, women’s employment status, religious affiliation, exposure to information, gestational age of pregnancy at first ANC booking, the number of ANC visits made before delivery, and the number of living children were statistically significantly associated with place of delivery. However, exposure to information and the number of living children were very strongly associated with home delivery. Comparatively, the odds of home delivery were higher for women not exposed to information (AOR = 13.64, p<0.001) and women with 2 (AOR = 4.64, p = 0.014), 3 (AOR = 4.96, p = 0.025), and 4 or more living children (AOR = 9.59, p = 0.001) (Table 3).

**Table 3:** Characteristics independently associated with home delivery among women aged 15–49 years in rural northern Ghana.

Characteristic	Adjusted Odds ratio	P-value
	AOR(95% CI)	
**Age (Years)**		
15–24	Reference	
25–34	1.06(0.39–2.86)	0.912
35–49	2.19(0.65–7.42)	0.206
**Maternal education**		
No formal education	0.61(0.09–3.95	0.602
Basic education	0.67(0.12–3.85)	0.654
Secondary or higher	Reference	
**Employment status**		
Unemployed	Reference	
Employed	0.09(0.03–0.24)	<0.001
**Religious affiliation**		
African traditional	Reference	
Islam	0.20(0.06–0.63)	0.006
Christian	1.24(0.55–2.85)	0.599
**Exposure to information**		
Exposed	Reference	
Not exposed	13.64(3.46–53.77)	<0.001
**Health insurance enrollment**		
Registered	Reference	
Not registered	2.16(0.80–5.82)	0.129
**Gestational age of pregnancy at first ANC booking**		
First trimester	0.04(0.01–0.14)	<0.001
Second trimester or over	Reference	
**Number of ANC visits before delivery**		
Less than 4	Reference	
4 or more visits	0.05(0.02–0.12)	<0.001
**Number of living children**		
1	Reference	
2	4.64(1.37–15.69)	0.014
3	4.96(1.22–20.12)	0.025
4 or more	9.59(2.50–36.89)	0.001

### Qualitative findings

#### Characteristics of women in the FGDs

There were 33 women in the discussions. Their ages ranged from 19 to 40 years. Sixteen of the participants had no formal education and only 4 had at least basic level education. All 33 women were married and living with their husbands at the time of the study. Nine women (27.3%) delivered their index child at home.

#### Available places for delivery by women in the study district

The women mentioned that two places were available in and around their respective communities for delivery. Women in the district used either the public health facilities which included the health centers, CHPS and hospital, or the home for delivery. The home deliveries were assisted by the TBAs. The narratives indicated that the participants were aware of the lifesaving opportunities associated with facility-based delivery.

*There are some women [TBAs] in the community who assist women to deliver but as for me I go to the health facility when I want to deliver (R5*, *Gbeenasa community)*

*There are TBAs in the community who help in delivery but at the hospital, people deliver and they do not have enough water [become dehydrated] or blood in the body and they [health staff] can transfuse. With the home deliveries, if there is dehydration or loss of blood, they do not have it to transfuse. That is why we go to the health facility. Because if you require any of these things*, *they [nurses] can give [transfuse] you water or blood (R9, Samsa community)*

*There is a midwife at the Doninga Health Center so when we are pregnant that is where we go to deliver*. *We do not have a midwife here, so anytime we want to deliver we go to the Doninga Health Centre (R3, Bachongsa community)*

#### Reasons why women deliver at home under the user-fees exemption policy in Ghana

The reasons why women delivered at home under the user-fees exemption policy in Ghana were explored in the FGDs. Four major themes emerged as perceived reasons for home delivery: (1) poor attitude of nurses; (2) lack of, and cost of transportation; (3) costs of delivery supplies; and, (4) traditional beliefs and practices (taboos).

#### Poor attitude of nurses (midwives)

Women hesitated, or avoided entirely, the use of health facilities for delivery due to the poor attitudes of nurses at the public health facilities. Participants described inappropriate treatment by midwives as a major barrier to utilizing skilled delivery. The women described witnessing or hearing stories of midwives shouting at women during delivery, being impatient, or negligent. On the other hand, women who delivered at home were comfortable with the support they received from family members.

*Some of them [expectant mothers] have said on several occasions that the nurses are not friendly; some of them [nurses] will shout at you and others [nurses] will maltreat you when you go there [health facility] to deliver (R4, Gbeenasa community)*.

*Some of the women say when they go to the health facility to deliver, there are some health staff who are not compassionate with the pregnant woman and will abandon you until you deliver on your own so there is no need going there [health facility] to deliver again. Some [expectant mothers] feel that if they deliver at home, their mothers and husbands will assist them to deliver safely (R3, Samsa community)*.

#### Lack of, and cost of transportation

The availability of transportation was a factor biasing women’s decision towards home delivery especially when labor begins at night. Although it became evident during the discussions that some of the selected communities had ‘rural ambulances’ (motor-king) situated within the community for referral purposes, women from the other communities had to rely on motorbikes from people within their network as their only available means of transportation to the nearest health facility. Nonetheless, women had to pay for the fuel (petrol) used which was also financially challenging. The following statements were recorded:

*For me, I will say it. Some of them [expectant mothers] say the labor started at night and they [expectant mothers] cannot walk in the night to the health facility to deliver. So, they will see if there is anyone in the community who can assist in the delivery (R1, Samsa community)*.

*Transportation is a major problem for pregnant women in this community because if you are in labor, there is no means of transportation to send you to the health facility to deliver unless you buy petrol into someone’s motorbike. If you do not have the money to buy petrol into the motorbike and you cannot also walk to the health facility, then it is obvious you will deliver at home*. *That is our main problem here [in the community] (R1, Gbeenasa community)*

*Even though this community has a motor tricycle, we have to buy petrol into it before it will carry us to the health facility for delivery. Unfortunately*, *the women here are poor and the men will not agree to use their money to buy fuel to transport us to the facility to deliver (R3, Bachongsa community)*

#### Cost of delivery kits

The data showed that despite the user-fees abolishment for maternal health services, significant costs remain in seeking facility-based delivery. Alongside, the cost of transportation, one commonly mentioned financial barrier was the cost involved in acquiring the “things” (i.e. sanitary pads, disinfectants, napkins, mackintosh, cloths for baby etc.) that expectant mothers were required to bring along with them to the health facility for delivery.

One woman lamented:

*When I was pregnant, I used to attend ANC regularly, but the buying of the items [Dettol, sanitary pad, mackintosh, etc.] for the delivery made me a victim [of home delivery]. I couldn’t buy the items so I didn’t go to deliver at the health facility (R7, Zamsa community)*.

*Some [expectant mothers] have given the testimony that they went to deliver at a health facility and after the delivery, the midwife asked her to give her [midwife] the pad she brought. When she failed to provide it, the midwife pushed her out to go home. She used her clothes to cover herself, but said that if she had delivered in the house this would not have happened to her. That is why some women refuse to go to the health facility. If they do not have the necessary items [Dettol, sanitary pad, mackintosh, etc.] they will not go to the facility to deliver. They say that if they go without them [Dettol, sanitary pad, mackintosh, etc.] the midwives will insult and humiliate them (R4, Gbeenasa community)*.

The narratives of the women further suggested that aside from these delivery kits, women sometimes have to purchase drugs from out-of-pocket due to the unavailability of the drugs at the visited health facility and this also placed a great deal of financial burden on them.

*When I was pregnant I visited the health facility but I was not in labor. They [nurses] prescribed drugs for us to go and buy [the drugs were not available at the facility visited]. On delivery too, they [nurses] asked me to buy Dettol antiseptic and a particular type of soap. All of these are financial challenges. I have to buy some for the health facility and also buy some for use when I return home from the health facility. Where do I get money to buy all these things? (R3*, *Bachongsa community)*

#### Traditional (cultural) beliefs and practices (taboos)

The findings showed that some families hold traditional beliefs and ceremonies that are perceived by the discussants to be barriers to utilizing delivery services provided by SBAs. It is a taboo for women from certain families to deliver in a health facility. Also, newborn babies must be presented to the ‘traditional gods’ before they are able to access essential newborn care services, including immunizations. There were perceived repercussions in the form of the death of either the woman or the child if the taboos were not adhered to. One woman commented in the FGDs:

*Some of them [families] also say pregnant women in their families will never go to the health facility to deliver. It is a taboo in their house. When you go to the health facility to deliver, either the mother, or the baby will die. Because I am afraid to die, do you think I will like to go [and deliver in the health facility] and die? (R1*, *Samsa community)*

Another woman confirmed:

*Yes, you will like to deliver at the facility but your in-laws will say that the women in their family do not deliver at the health facility so they will not allow you to go. They claim it is a taboo in their family so you cannot go to a health facility to deliver. They threaten you that if you disobey their customs you will end up dying at the facility when you go to deliver. You will, therefore, have to deliver at home. After the delivery, they will have to present the child to the “gods” before you can go to the health facility for child welfare care services (R6, Bachongsa community)*.

## Discussion

This study used both quantitative and qualitative research methodologies to determine the prevalence of home delivery, the characteristics of women delivering at home and the reasons for having home deliveries under the free maternal health care policy in a rural district in the northern part of Ghana. The quantitative results showed that 38% of deliveries in the study area occurred at home. Although this percentage is lower than the 58–79% reported for rural women from other LMICs such as Bangladesh, Ethiopia, Tanzania and Kenya [29–32], it is a concern for the achievement of the SDGs of reducing maternal and infant mortality globally. The findings also affirm the existing documentation that some women in SSA use health facilities for ANC but go on to deliver at home where SBAs are more likely to be absent [13,32]. Notwithstanding, we believe that among other maternal health interventions in the study area, the re-orientation of TBAs in the Upper East region of Ghana to referring or accompanying pregnant women to the health facilities to deliver, instead of conducting the deliveries themselves, might have contributed to the relatively lower proportion of home deliveries noted in this study [33,34].

Our findings are consistent with previous studies which reported that women without exposure to information and women with at least two living children were more likely to deliver at home [30–32]. More importantly, our findings showed that health workers and community health volunteers are important sources for information on maternal health in rural areas and can be utilized to disseminate maternal health information in these settings. Exposure to maternal health information increases women’s knowledge and understanding of maternal health issues. A study among Ethiopian women reported that women who delivered at home had inadequate knowledge of maternal health services [12]. Health education and promotion activities are known to have significant impact on poor and less educated women [35]. Therefore, health education activities advertising the use of maternal health services, most especially the use of health facilities for delivery services, are important determinants for their acceptability and utilization in rural areas [36]. Moreover, women are more likely to deliver in health institutions if they are advised to do so by health workers.

Nevertheless, the qualitative findings of this study provide an insight into the contextual factors defining home delivery by rural mothers under the user-fees exemption for the utilization of maternal health services in Ghana. This study illustrates that there are other health systems, as well as environmental, and social-cultural factors that need to be addressed to improve acceptability and use of facility-based delivery services.

Firstly, the poor attitude of health care workers characterized by nurses’ lack of patience and use of abusive language towards women who come to deliver in public health facilities was identified as a key barrier to the use of delivery services by the women in this study. Several studies have demonstrated that staff attitude towards expectant mothers plays an important role in influencing their acceptability and use of maternal health services. Two previous studies in Ghana provided proof that the interpersonal aspects of the health care system are important determinants of women’s expectations, which in turn influence their satisfaction and current or subsequent use of the system [37,38]. Similar observations were made in Nigeria where despite the high utilization of prenatal care services, two in five women ended up delivering at home, and the unfriendly attitude of health care workers was the second most important reason for women’s preference for the home for delivery [13]. Furthermore, the TBAs are perceived to be respectful, friendly, and trustworthy by women who choose home delivery [39].

Secondly, women’s recourse to home delivery is influenced by the lack of transportation to convey them to the nearest maternal health facility for delivery. Geographical distance and long travel hours have long been recognized as barriers to the use of maternal health services [13,17,31,32]. Also, consistent with findings from this study, earlier studies have confirmed that labor can occur at any time of the day, and due to the lack of transport, women end up delivering at home [29]. The Sustainable Emergency Referral Care (SERC) initiative was a component of the 6-year Ghana Essential Health Intervention Program (GEHIP), a health systems strengthening initiative designed to promote access to health care in northern Ghana [40]. The SERC was designed as a low-cost emergency transportation system to address access barriers to emergency care services. Under this system, patients are transported from their communities to higher levels of care using a customized module of the three-legged vehicle well known as motor-king [41]. During the 2-year pilot period, the operational costs of the SERC were supported by the Ghana Health Service (GHS). To encourage facility-based delivery, normal labor cases were transported free of charge. Our findings showed that some of the communities selected were beneficiaries of the motor-king ambulance. However, users, including laboring women are now obliged to pay for the cost of the fuel in order to use the service. Some women could not afford the cost of fuel and, therefore, had recourse to home delivery. In other instances, such as when a motor-king was not available in the community, some women relied on the services of motorbikes owned by neighbors which are not suitable for transporting pregnant women to the health facility. Expectant mothers’ reliance on people within their social networks for logistical support in accessing the health facility does not always result in a safe and timely arrival for delivery [42]. The lack of money for transport has also been observed to influence home deliveries by women in Zambia [39]. These findings suggest that there is an interplay of many factors in determining access to maternal health services in deprived communities.

Another perceived barrier to health facility delivery was women’s inability to acquire the “items” needed for delivery in health institutions. Women who resort to home delivery are discouraged by the expensive supplies, including sanitary pads, disinfectants, napkins, and mackintosh which they are mandated to bring along for delivery at the health facility. The inability of expectant mothers to acquire these supplies and the concomitant humiliation by the midwives made seeking health facility delivery prohibitive. Bazzano and his colleagues elucidated that skilled delivery incurs financial costs in the acquisition of delivery supplies and, for this reason, rural women without financial capability prefer to deliver at home [43]. Consistent with our findings, a study in Zambia identified that the lack of funds for baby clothes and requirements for the mother during and after labour were barriers to the use of health facilities for delivery services by some women [39]. From our perspective, the idea of the Ghana free maternal health care policy was to reduce financial costs and improve the access to and use of skilled delivery services. However, some medical supplies are not covered under this policy and our findings showed that acquiring these medical supplies places a financial burden on families. Therefore, integrating these supplies into the policy cannot be overemphasized.

Finally, the study revealed that the low or non-utilization of maternal health services is due, in part, to existing traditional beliefs and practices or taboos. Many communities in the upper areas of northern Ghana appear to have been enlightened by public health campaigns, which in turn has led to a gradual shift in social norms from home delivery in favor of delivery by SBAs [42,44]. Regardless of this, our findings revealed that although women acknowledged that there are lifesaving benefits associated with facility-based delivery, pregnant women from some households were not permitted to deliver in a health facility due to some traditional beliefs and practices. Pregnant women who failed to abide by such traditions are met with serious repercussions including the death of the woman or the baby. Furthermore, the baby must be presented to the ancestral gods before the woman can utilize child welfare services. Negative traditional practices are not limited to only rural settings in Ghana. In rural Cambodia for instance, ‘roasting’ is the most popular tradition and women who deliver at home do it for the opportunity to be roasted: “I delivered at home so that I could be roasted at home”[45]. This practice requires that after delivery, a woman must spend at least three days, and at most one month, lying beside a fire or on a mat bed over coals where she is “roasted”, in order to regain the strength and heat that are perceived to be lost during delivery. However, these negative traditional beliefs and practices can be harmful to women and children’s health.

There are some caveats about our study which need to be noted. The study used a cross-sectional design and, therefore, only inferences can be deduced. In addition, the study was conducted in a predominantly rural setting, thus the findings may differ in urban settings. However, using qualitative research alongside quantitative methodology enabled us to explore the reasons behind women’s choice of home delivery from their own perspectives which could not have been fully understood using only the quantitative methods. The FGDs have some quality controls and reliability checks, in terms of the information provided, as the participants tended to balance their views with those of others, enabling participants to express their views on the topic and generating additional areas of discussion.

## Conclusion

Despite the government's efforts to offer free maternal health services in Ghana, a significant proportion of women still deliver at home. The findings showed that socio-demographic and obstetric factors play an important role in influencing women’s use of skilled delivery services, as do health system and socio-cultural factors. Addressing the ‘hidden costs’ to health facility delivery, including integrating the cost of delivery kits into the free maternal health care policy, addressing the negative attitude of health staff towards clients, improving the transportation networks, and creating awareness on the negative traditional beliefs and practices that are consequential to maternal health may contribute to improving the use of health facilities for delivery services by rural women in Ghana.

## Supporting information

S1 File Study questionnaire for women aged 15–49 years. (PDF)

S2 File Focus group discussion guide for women aged 15–49 years. (PDF)

S3 File The thematic analysis steps used to analyze the qualitative data. (PDF)
