# Supplementary material for: “I couldn’t buy the items so I didn’t go to deliver at the health facility” Home delivery among rural women in northern Ghana: A mixed-method analysis
Source: PLoS One. 2020 Mar 12;15(3):e0230341. doi: 10.1371/journal.pone.0230341 (PMC7067411; doi:10.1371/journal.pone.0230341)
Supplement: S1 File — (PDF) [file pone.0230341.s001.pdf]

Questionnaire for women aged 15-49 years in quantitative study

Compound name:…………………………Community name………………………...

Date of interview………….

No	Question	Code
	**SOCIO-DEMOGRAPHIC DATA**	
1	**Age of respondent** (in completed years)……… (confirm from any valid ID)	Q1age
2	**Marital status**<br>Single……………………………………………………….……..1<br>Married……………………………………………………….…...2<br>living with partner ………..……………………………………...3<br>Divorce/separated……………..………………………………..4<br>Widow……………………….……………….....................5<br>**(CIRCLE ONLY ONE)**	Q2mar_st
3	**Respondent's occupation**<br>Unemployed………..…………………………………………1<br>Farmer…………………………………………………………...2<br>Trader/Business………………………………………………….3<br>Government employee…………………………………………...4<br>Private sector employee…………………………………………5<br>Others (*specify)*..………………………………………………<br>**(CIRCLE ONLY ONE)**	Q3occup
4	**Educational level (highest level attained)**<br>No education……………………………………………………1<br>Primary ………………………………………………………….2<br>Middle/JHS……………………………………………………….3<br>SHS………………………………………………………………4<br>Tertiary and above……………………………………………….5<br>**(CIRCLE ONLY ONE)**	Q4educ
5	**Religion**<br>Traditional………………………………………………………..1<br>Christian…………………………………………………………2<br>Moslem…………………………………………………………...3<br>Others *(specify)*..………………………………………………<br>**(CIRCLE ONLY ONE)**	Q5relig
6	**Parity**<br>1…………………………………………………………………..1<br>2…………………………………………………………….….2<br>3…………………………………………………………………..3<br>4+………………………………………………………………4<br>**(CIRCLE ONLY ONE)**	Q6part
7	**Ethnicity**<br>Builsa……………………………………………………………1<br>Kantoosi…………………………………………………………2<br>Mamprusi………………………………………………………...3<br>Kassena ………………………………………………………….4<br>Sissala……………………………………………………………5	Q7ethnic

	Others (*Specify)*…………………………………………….………<br>**(CIRCLE ONLY ONE)**	
8	**Where you registered to the National Health Insurance Scheme in your last pregnancy? *(If yes ask to see card)***<br>Yes……………………………………………………………1<br>No…………………………………………………………. ...2 → **(CIRCLE ONLY ONE)**	Q8insura<br><br>**SKIP TO 10**
9	**Health insurance status (as at last pregnancy)**<br>Invalid health insurance…………………………………………1<br>Valid health insurance……………………………………………..2<br>Not applicable……………………………………………………..88<br>**(CIRCLE ONLY ONE)**	Q9valdins
	**KNOWLEDGE ON BENEFITS OF ANC AND USE OF ANC**	
10	**Did you attend ANC for your most recent child [name]?**<br>Yes…………………………………………………………..1<br>No……………………………………………………………...2 →<br>**(CIRCLE ONLY ONE)**	Q10anc_at<br><br>**SKIP TO 16**
11	**At what age (in months) of pregnancy did you start ANC?(*comfirm from ANC record book)***<br>1month………………………………………………………1<br>2 months…..……………………………………………………2<br>3months…………………………………………………….....3<br>4 months…………………………………………………….4<br>5months……………………………………………………..5<br>6months……………………………………………………...6<br>7months and above…………………………………………7<br>Not applicable………………………………………………88<br>**(CIRCLE ONLY ONE)**	Q11gest
12	**Where did you receive ANC services for [name of recent child]? (probe) (Confirm from ANC book)**<br>Health centre………………………………………………1<br>CHPS……………………………………………………….2<br>TBA………………………………………………………..3<br>Others *(specify)*……………………………………………….<br>Not applicable……………………………………………..88<br>**(CIRCLE ALL THAT APPLY)**	Q12wheranc
13	**How many visits (excluding visits for own medical care) did you make before delivering [name]?(*confirm with ANC book)***<br>1……………………………………………………………..1<br>2……………..…………………………………………………2<br>3……………………………………………………………..3<br>4……………………………………………………………..4<br>5 and more…………………………………………………..5 →<br>Not applicable………………………………………………88<br>**(CIRCLE ONLY ONE)**	Q13visits<br><br>**SKIP TO 15**
14	**What is/are your reasons for making this number of visits?(*refer to 13)*(probe)**<br>Health facility too far…………………………………………..1<br>No transport to health facility..………………………………...2<br>Cost of ANC………………………………………………..3	Q14reas

	I did not see the need……………………………………………..4<br>Poor attitude of health workers………………………………..5<br>Long waiting time at facility…………………………………..6<br>Others (*Specify*)……………………………….……………….<br>Not applicable…………………………………………………88<br>**(CIRCLE ALL THAT APPLY)**	
15	**Why did you attend ANC? (probe)**<br>To check my health and baby's health…………………………1<br>To see if baby is lying well…………………………………….2<br>To receive immunizations………………………………………3<br>Advice from health workers ……….…………………………..4<br>Good quality of care……………………………………………5<br>Others *(specify*..…..…………………………………………….<br>Not applicable…………………………………………………88<br>**(CIRCLE ALL THAT APPLY)**	Q15whyanc
16	**Why didn't you attend ANC? (probe)**<br>I did not see the need for ANC………..……………………1<br>I had no complication with pregnancy…………..…………..2<br>Unintended pregnancy……………………………………….3<br>No money……………………………………………………4<br>Health facility too far………..……………………………….5<br>Poor attitude of health workers…………………………………6<br>Medicines given at health facility are not good………………..7<br>Others *(specify)*..……………………………………………..	Q16noanc
	**(CIRCLE ALL THAT APPLY)**	
17	**In your opinion at what age of pregnancy should a woman start ANC?**<br>1month……….…………………………………………………..1<br>2 months…..……………………………………………………..2<br>3months…………………………………………………………..3<br>4 months………………………………………………………4<br>5months…………………………………………………………..5<br>6months…………………………………………………………..6<br>7months …………………………………………………………7<br>8 months and above……………………………………………8<br>**(CIRCLE ONLY ONE)**	Q17oppanc
18	**What is your reason(s) for your answer? (probe)**<br>There is no need for ANC……………………………………1<br>No problem/complication with pregnancy……………………..2<br>Unintended pregnancy……………………………………….3<br>No money……………………………………………………4<br>Health facility too far……..……………………………………..5<br>Poor attitude of health workers…………………………………6<br>Medicines given at health facility are not good………………..7<br>Others (*Specify)*..…...……………………………………….. **(CIRCLE ALL THAT APPLY)**	Q18oppreas
19	**In your opinion why should a pregnant woman attend ANC? (probe)**<br>To check my health and baby's health…………………………1<br>To see if baby is lying well…………………………………….2<br>To receive immunizations……………………………………...3	Q19oppatt

	Advice from health workers and others………………………..4<br>Good quality of care……………………………………………5<br>Husband's decision……………………………………………..6<br>Others *(specify)*..……………………………………………….<br>**(CIRCLE ALL THAT APPLY)**	
20	**In your opinion why would a pregnant woman <u>NOT</u> attend ANC? (probe)**<br>No need for ANC………………………………………………1<br>No problem/complication with pregnancy……………………..2<br>Unintended pregnancy…………………………………………3<br>No money………………………………………………………4<br>Far distance to health facility…………………………………..5<br>Poor attitude of health workers…………………………………6<br>Others *(Specify)*..………………………………………………..<br>**(CIRCLE ALL THAT APPLY)**	Q20oppno
21	**In your opinion why do some women start ANC late (that is after 3 months)? (probe)**<br>No need for early initiation……………………………………..1<br>Unaware of pregnancy…………………………………………2<br>No complications with previous pregnancy…………………….3<br>No money for ANC……………………………………………4<br>Husband's decision not to let her go for ANC..………………..5<br>Health facility too far…………………………………………..6<br>Lack of transport to health facility……………………………..7<br>Poor attitude of health workers…………………………………8<br>Medicines given at health facility are not good………………..9<br>Others *(specify)*..………………………………………………..<br>**(CIRCLE ALL THAT APPLY)**	Q21opplate
	**DELIVERY CARE**	
22	**Where did you deliver [name]?**<br>Health facility…………………………………………………1<br>Home……………………………………………………………..2<br>Others *(specify)*................……………………………………<br>**(CIRCLE ONLY ONE)**	Q22delvr<br><br>**SKIP TO 24**
23	**Why did you deliver [name] at a health facility? (probe)**<br><br>Safer delivery…………………………………………………..1<br>Advice from health workers and others………………………..2<br>Previous complications with child birth……………………….3<br>Quality care……………………………………………………4<br>Husbands decision……………………………………………..5<br>Others (*specify)*…..……………………………………………<br>Not applicable…………………………………………………88<br>**(CIRCLE ALL THAT APPLY)**	Q23hfdelvr
24	**Why did you deliver [name] there? (probe)**<br><br>Safer delivery…………………………………………………1<br>Advice from mother-in-law……………………………………..2<br>Previous complications with child birth………………………...3<br>Quality care……………………………………………………..4	Q24hmdelvr

	Cheaper cost for services……………………………………….5<br>Husbands decision…………………………………………....6<br>Only available option…………………………………...7<br>Others (*Specify*)……………………………………………<br>**(CIRCLE ALL THAT APPLY)**	
25	**In your opinion why would a woman <u>DELIVER</u> in a health facility? (probe)**<br>Safer delivery……………………………………………………1<br>Advice from health workers and others………………………….2<br>Previous complications with child birth…………………………3<br>Quality care……………………………………………………4<br>Husbands decision………………………………………………5<br>Only available option……………………………………………6<br>Others (*Specify*..………………………………………………….<br>**(CIRCLE ALL THAT APPLY)**	Q25ophfdel
26	**In your opinion why would a woman <u>NOT DELIVER</u> in a health facility? (probe)**<br>No problem/complication with pregnancy……………………..1<br>Unaware of delivery……………………………………………2<br>No money………………………………………………………3<br>Husband's decision……………………………………………4<br>Distance to health facility………………………………………5<br>Poor attitude of health workers…………………………………6<br>Poor quality of care at health facility…………………………..7<br>Others *(specify)*..………………………………………………..<br>**(CIRCLE ALL THAT APPLY)**	Q26opndel
27	**What are the benefits from delivering in a health facility? (probe)**<br><br>Safer delivery……………………………………………………1<br>Quality care for mother and baby………………………………..2<br>Good medicines…………………………………………………3<br>Others (*Specify*)…………………………………………………...<br>**(CIRCLE ALL THAT APPLY)**	Q27benhf
**EXPOSURE TO INFORMATION ON MATERNAL HEALTH**		
28	**Where do you hear information on ANC utilization and delivery care? (probe)**<br><br>Radio……………………………………………………………1<br>TV………………………………………………………………2<br>Health workers…………………………………………………3<br>Community volunteers…………………………………………4<br>Others (*Specify*)…………………………………………………<br>I don't hear information on maternal health……………………5 ⟶<br>**(CIRCLE ALL THAT APPLY)**	Q28mhinfo<br><br>**END INTERVIEW**
29	**How often do you hear information on ANC and delivery?**<br>Daily ……………………………………………………………1<br>Weekly……………………………………………………………2<br>less than 3times a month……………………………………..3<br>more than 4 times in a month…………………………………..4<br>yearly ……………………………………………………………..5	Q29hwoftn

	Only mass campaigns……………………………………………..6<br>Others (*Specify*) ……………………………………………….<br>Not applicable………………………………………………..88<br>**(CIRCLE ONLY ONE)**	
30	**What does the information say or cover? (probe)**<br><br>Sleep under insecticide net every night during pregnancy……..1<br>Attend ANC early……………………………………………2<br>Deliver in health facilities……………………………………3<br>Observe personal and environmental hygiene…………………4<br>Send children for CWC monthly………………………………5<br>Exclusive Breastfeeding ………………………………………6<br>Others (*specify)*………………………………………………..<br>Not applicable…………………………………………………88<br>**(CIRCLE ALL THAT APPLY)**	

Thank participant for her time
