# Supplementary material for: “I couldn’t buy the items so I didn’t go to deliver at the health facility” Home delivery among rural women in northern Ghana: A mixed-method analysis
Source: PLoS One. 2020 Mar 12;15(3):e0230341. doi: 10.1371/journal.pone.0230341 (PMC7067411; doi:10.1371/journal.pone.0230341)
Supplement: S2 File — (PDF) [file pone.0230341.s002.pdf]

FOCUS GROUP DISCUSSION GUIDE FOR WOMEN AGED 15-49 YEARS
Guide for Moderator
Welcome the participants and introduce yourself and the note taker. Ask participants to introduce themselves. Explain that the note taker is available to record detailed notes during the discussion. Explain that the study is for academic purposes and the aim is to gain an in-depth understanding of the practices and experiences regarding maternal health in their community, especially issues relating to antenatal care and delivery. You hope that their responses to the questions will be important in understanding the situation and the knowledge gained will be shared with the District Health Team to help to improve maternal health care in their area. In future, the results will be published so that other researchers can also learn from them. The discussion will last about 60-90 minutes and because of the exhaustive nature of the discussion, you will ask their permission to record the discussion to be certain that you have captured their views fully. Explain that you will like to assure them of confidentially and to this end you will assign to them pseudonyms (show cut card boards with numbers) which they will use throughout the discussion as their names. Also explain that whatever that will be discussed should not be shared with anyone outside the group. Ask if anyone has a question to ask about the information communicated or the study before you can proceed. Ensure informed consent forms have been signed or thumb printed by participants. Ask permission to turn on the recorder.

Breaking the ice
First, I would like to ask you some questions about your community

1. What are the major health care problems of this community?
2. What problems affect only women? (Probe)

Now we are going to talk about antenatal care practices in your community

DISCUSSION ON ANC PRACTICES

- At what period of pregnancy do women in this community start attending ANC?

    - Ask participants to share their own experiences

- What do you think is the best time to begin antenatal care? Why? [probe]
- Where do women in this community normally go to receive care when they are pregnant? [probe]
- Why do women in this community seek antenatal care? [probe]
- Why do some women in this community start ANC late? [probe]
- What are the benefits of going to the health facility early (rather than late) in pregnancy to seek ANC? [probe]
- What happens at the health facility when you visit for ANC? [probe]
    - Ask participants to share their experiences

DISCUSSION ON DELIVERY PRACTICES

We are now going to talk about delivery practices in your community

- Where do most women in this community deliver?
- What other delivery places do women use in this community? [probe]
- Why do some women in this community deliver at the health facility? [probe]

- What happens at the health facility when you go to deliver? [probe]
    - Ask participants to share personal experiences

- Why do some women in this community deliver at home and not at the health facility?[probe]
    - find out from among the women those who delivered at home and ask them to share personal reasons for delivering at home
- What are the problems that women in this community face during labor and delivery? [probe]
- How do you think these problems can be solved?[probe]

MATERNAL HEALTH INFORMATION

- Where do women in this community hear information on antenatal care? [probe]
- How often do women in this community hear health information on antenatal care? [probe]
- What is the information always about? [probe]
- Where do women in this community hear information on delivery care? [probe]
- How often do women in this community hear health information on delivery care? [probe]
- What is the information always about? [probe]

We have come to the end of our discussion. Is there anything that you will like to add to the discussion?

Thank participants for their participation
