# Supplementary material for: “I couldn’t buy the items so I didn’t go to deliver at the health facility” Home delivery among rural women in northern Ghana: A mixed-method analysis
Source: PLoS One. 2020 Mar 12;15(3):e0230341. doi: 10.1371/journal.pone.0230341 (PMC7067411; doi:10.1371/journal.pone.0230341)
Supplement: S3 File — (PDF) [file pone.0230341.s003.pdf]

The following steps were followed to generate codes and develop themes:

a) Familiarization

The transcripts from the FGDs were read several times to have an understanding of the content of the data. Ideas and patterns relevant to the study objectives identified during the reading process were written down.

b) Coding

After familiarizing with the data, we produced initial codes by organizing and documenting ideas and patterns within concepts described in the data on the margins of the transcripts. These codes are labels attributed to specific sections of the transcripts including paragraphs or sentences to aid in the systematic arrangement of important concepts without altering the context in which these concepts appear. All the codes and relevant data extracts on delivery were collated and used in the later stages of the analysis.

c) Searching for themes

Relevant themes on delivery practices important to the research objectives were documented to attain some patterned meaning within the data set given. The codes and collated data on delivery were examined by the researchers to identify significant broader patterns of meaning by sorting and categorizing recurrent and unifying codes. The data relevant to each candidate theme were collated and reviewed for the viability of each candidate theme

d) Reviewing themes

The potential themes were refined into real themes and all the extracts under each theme were read thoroughly to identify any coherent pattern. This ensured that there was enough data to support each potential theme. During the refinement process, separate themes that were unifying were collapsed to form one theme.

e) Defining and naming themes

Detailed analysis was carried out on the themes to identify the "story" told by each theme in relation to the research objectives. We identified and determined the aspect of the data captured by each theme by paraphrasing responses from respondents in the data extracts and applied quotations where necessary.
